# Solar Radiation Exposure and Outdoor Work: An Underestimated Occupational Risk

**DOI:** 10.3390/ijerph15102063

**Published:** 2018-09-20

**Authors:** Alberto Modenese, Leena Korpinen, Fabriziomaria Gobba

**Affiliations:** 1 Department of Biomedical, Metabolic and Neural Sciences, University of Modena & Reggio Emilia, 41124 Modena, Italy; fabriziomaria.gobba@unimore.it; 2 Clinical Physiology and Neurophysiology Unit, The North Karelia Central Hospital, 80210 Joensuu, Finland; leenakorpinen@gmail.com

**Keywords:** solar radiation, ultraviolet radiation, skin cancers, macular degeneration, cataract, occupational exposure, outdoor work

## Abstract

A considerably high number of outdoor workers worldwide are constantly exposed for the majority of their working life to solar radiation (SR); this exposure is known to induce various adverse health effects, mainly related to its ultraviolet (UV) component. The skin and the eye are the principal target organs for both acute and long-term exposure. Actinic keratosis, non-melanoma skin cancers, and malignant melanoma are the main long-term adverse skin effects, whereas in the eye pterygium, cataracts, and according to an increasing body of evidence, macular degeneration may be induced. Despite this, SR exposure risk is currently undervalued, if not neglected, as an occupational risk factor for outdoor workers. SR exposure is influenced by various environmental and individual factors, and occupation is one of the most relevant. For a better understanding of this risk and for the development of more effective prevention strategies, one of the main problems is the lack of available and adequate methods to estimate SR worker exposure, especially long-term exposure. The main aims of this review were to provide a comprehensive overview of SR exposure risk of outdoor workers, including the UV exposure levels and the main methods recently proposed for short-term and cumulative exposure, and to provide an update of knowledge on the main adverse eye and skin effects. Finally, we also outline here preventive interventions to reduce occupational risk.

## 1. Introduction

Solar radiation (SR) exposure can possibly be considered the oldest occupational risk. The exposure started together with the first human activities as hunting and fishing and then agriculture. The adverse effects of this risk were recognized and described by Bernardino Ramazzini in 1700 in his textbook “*De Morbis Artificum Diatriba*”, reporting of naked farmers with extensive sunburns and associated hyperthermia [1]. As an occupational risk, SR can be considered a physical risk; the sun emits a wide range of the frequencies of the whole electromagnetic spectrum, but mainly in the range of non-ionizing radiations (NIR), including most of the natural incoherent optical radiation. According to the International Commission on Illumination (Commission Internationale d’Eclairage—CIE) [2], the spectrum of the optical radiation can be classified as:

(1) Ultraviolet radiation (UVR), composed of UV-C (wavelength; λ = 100–280 nm), UV-B (λ = 280–315 nm), and UV-A (λ = 315–400 nm);
(2) Visible radiation (λ = 400–780 nm);
(3) Infrared radiation (IR), further divided into IR-A (λ = 780–1400 nm), IR-B (λ = 1400–3000 nm), and IR-C (λ = 1 mm–3000 nm).

Generally speaking, optical radiation has a limited penetration depth in biological tissues. However, each spectral region is different, both in terms of penetration ability and biological interaction mechanisms, and consequently in terms of possible induced effects [3].

The solar radiation spectrum at the Earth’s surface is quite different from that emitted by the Sun due to the shielding effect of various atmospheric components. Ozone is particularly important as it filters out all the wavelengths shorter than 290 nm, i.e., all the UV-C and the majority of UV-B [4]. About the 95% of the solar radiation at the Earth’s surface is composed by IR and visible wavelengths, at about 45% and 50%, respectively; whereas UV accounts for approximately 5%. Nevertheless, UVR is able to induce the main and most severe adverse health effects on humans, and is responsible for the classification of SR in Group 1 of the International Agency for Research on Cancer (IARC), which includes agents carcinogenic to humans [5]. Furthermore, the depletion of the ozone layer over the last decades has increased the amount of UV-B at the Earth’s surface. For these reasons, in this paper, the health risks associated to solar UVR exposure are mainly considered.

According to these premises, the main objective of this review is to provide a comprehensive overview of occupational SR exposure risk of outdoor workers. In the following sections, we offer a complete panorama of this risk from an occupational medicine point of view: the mechanisms through which UV rays interact with the biological tissues, the characteristics of exposure, and the usual UV levels reported in scientific literature for outdoor workers. Furthermore, an updated summary of the possible eye and skin adverse health effects related to occupational SR exposure in outdoor workers is presented, including results from recent systematic reviews. Finally, we outline here also the main preventive measures applicable to reduce solar UV occupational risk to better protect outdoor workers.

## 2. Solar Radiation: Mechanisms of Interaction and Pathophysiology

The interaction of solar radiation with biological tissues is related to two main mechanisms: photochemical, typical of the ultraviolet wavelengths, and thermal, the main mechanism in the case of infrared radiation. In the visible region of the SR spectrum, both mechanisms can be observed: the photochemical effect prevails in the violet-blue light region of the visible spectrum (400–550 nm wavelength), whereas the thermal effect prevails in the yellow-red light part (600–700 nm) [4,6].

Considering the thermal effect, when the photons are absorbed, they increase the kinetic energy of the tissue and the radiant energy of the optical radiation is converted to heat. The resulting rise in temperature depends on the wavelength, exposure duration, and total energy absorbed by the tissue. If the thermal energy is sufficiently high, other biological effects can be observed, for example, on procollagen expression with a consequent increasing in the production of reactive oxygen species (ROS). These effects have a threshold that can be rapidly exceeded in the case of high IR exposure levels, which are usually related to artificial sources. Moreover, the thermal sensitivity of different tissues is highly variable, mainly depending on dissipative processes related to the area of the irradiated surface and the composition of the tissue [6].

Photochemical effects, typical of UVR and of shorter wavelengths of visible radiation as violet-blue, are essentially related to the absorption of photons by specific molecules in target tissues, including DNA, called chromophores. The effects related to the photochemical mechanism depend on the total dose, such as a result of the product between the duration of the exposure and the intensity of the radiation. Accordingly, high short-term exposure and less intense but more prolonged exposure can induce similar effects [4]. This characteristic is known as the Principle of Reciprocity of Photobiology, or Bunsen-Roscoe Law of Photobiology [7].

The main target organs of the effects induced by SR are the skin and eyes. For both, the effects depend primarily on the wavelength, which characterizes the mechanism of interaction and the penetration level into biological tissue. During photochemical interactions, this is important for the presence and location of the interacting chromophores.

Regarding the skin, the three main layers—the epidermis, dermis, and hypodermis (also called subcutaneous layer)—have different specific components of epithelial, mesothelial, and neural origin. Longer UV wavelengths penetrate deeper. The stratum corneum provides an optical barrier for the UVC, whereas up to 25–50% of UVA can reach melanocytes in the dermis. In general, visible wavelengths and IR, particularly IRA, penetrate deeper into the skin, even reaching the hypodermis. Conversely, the penetration of IRC is limited to the epidermis.

In epidermal cells, solar UVR is absorbed from various chromophores of the cytosol and of cell membranes, including DNA and RNA. These interactions are due to the formation of reactive products, such as free oxygen radicals. In the case of DNA damage, this may induce skin cell divisions, contributing to the thickening of the epidermis. Upon acute irradiation, several cytokines are liberated, activated, or synthesized by keratinocytes. They belong to several categories and are responsible for local or systemic inflammatory reactions, such as vasodilatation, edema, and possibly hyperpyrexia [4,8,9,10]. The peak of UV absorption from DNA occurs at an about 260 nm wavelength; then, the absorption of UV-B rays with longer wavelengths rapidly decreases, and no absorption is detected for UV rays with wavelengths longer than 325 nm. DNA damage may result both from direct absorption of UVR and from oxidation due to the action of reactive oxygen species (ROS). A simple irradiation UV dose of approximately 1 Minimal Erythemal Dose (MED) results in about 300,000 DNA lesions per cell, most of them being repaired within a few hours [4]. Among the best known and most frequent DNA alterations related to solar UVR is the formation of cyclobutane-type pyrimidine dimers. The 6-4 pyrimidine dimers are often identified UV-induced DNA alterations. Considering UV-A, DNA photo-damage is more indirect and consequent to oxidation, which induces alterations based on the UV absorption in specific chromophores called 8-hydroxydeoxyguanosine (8-OHdG) and on the formation of DNA-protein crosslinks [4,8,9,10].

The main skin reactions to solar ultraviolet radiation exposure are neo-melanogenesis and skin thickening, which are responsible for skin darkening (tanning), which can be interpreted as an adaptive defense mechanism. Long-term exposure induces the complex phenomenon of photo-ageing, related to different UV components, but mainly to chronic UV-A damage [8,9,10].

Also, in the human eye, different SR bands are absorbed by different ocular structures, and consequently, different thermic and photochemical effects are possible. UV-C is absorbed by the cornea, whereas UV-B and UV-A rays are absorbed by the cornea and the lens, respectively. Notably, approximately 1–2% of near UV-A (380–400 nm) can reach the retina; age related differences have been described with proportions up to 10% in childhood. The whole visible spectrum and near infrared (IR-A) are absorbed by the retina, whereas the IR-B from the cornea and the lens and the IR-C from the cornea [4,6,11,12,13].

Even if adaptation mechanisms, such as pigmentation and thickening as described for the skin, are not available in the eye, other factors, such as the conformation of the frontal and orbital bones, provide an effective defense for the eye from overhead SR exposure. Other important defense mechanisms in case of intense direct light reaching the eyes are squinting and aversion responses, which are immediate involuntary responses appearing in fractions of second as an adaptation to sudden changes in lighting, even if some recent experiments with lasers showed that a significant fraction of the population may be not adequately protected by these responses [14]. Another mechanism is pupillary light reflex, which is the regulation of the pupil diameter in relation to the light intensity, is slower and is scarcely efficient in some neuro-ophthalmological conditions (anisocoria, myasthenia, paralysis of the ocular nerves, etc.) or in case of consumption of drugs and narcotics such as atropine and cannabis [4,6,11,12,13].

## 3. Main Factors Influencing Eye and Skin Solar Ultraviolet Radiation Exposure

### 3.1. Environmental Factors

According to the International Commission on Non-Ionizing Radiation Protection (ICNIRP), the main environmental factors influencing both the total amount and the spectral composition of solar UVR reaching the earth’s surface are [4]:

(1) Atmospheric composition: In addition to ozone, other gaseous and pollutants particles in the atmosphere may interact with UV rays, inducing various optic phenomena, such as absorption, reflection, refraction, and diffusion. The presence of pollutants in the troposphere usually reduces UVR exposure, but these phenomena can also increase the exposure in particular cases.
(2) Angle of the sun on the horizon, which depends on:
  - Hour of the day: In summer, about 20–30% of the total exposure to UVR occurs between 11:00 a.m. and 1:00 p.m., and 75% between 9:00 a.m. and 3:00 p.m. Season: In temperate countries, there are significant seasonal variations in exposure, whereas these changes are smaller closer to the equator.
  - Latitude: The cumulative UVR exposure decreases with increasing distance from the Equator.
(3) Altitude: UVR exposure increases with altitude. Approximately every 300 m, the solar UV ability in inducing sunburns increases 4%.
(4) Clouds: Solar UVR is approximately reduced by 50% with complete cloud cover, whereas incomplete coverage is not able to adequately shield UVR, where only 10% is usually blocked by the clouds. In some cases, diffusion, refraction, and reflection phenomena can even increase the amount of UVR.
(5) Reflectance: Reflection of surrounding surfaces can be relevant for individual solar UV exposure, possibly increasing the exposure of parts of the body usually protected from direct UV-rays, such as the eyes. Reflectance is high for white or clear surfaces, such as fresh snow, reaching values in the order of 0.8–0.9, whereas grass and foliage reflects only about 2% or less of the UVR, and sand reflects up to 15–20%. The reflection of water depends on various factors, including the sun angle, ranging from less than 10% to 65% or more in case of very low angle on the horizon. Another term used to describe this phenomenon is “albedo”. A particular aspect of albedo is the “Coroneo effect”: the rays coming from the temporal side of the face can be refracted by the corneal dome in the nasal corneal limbus and in the nasal and inferonasal part of the lens [4,11].

### 3.2. Individual Factors

The other group of factors influencing solar UV exposure includes personal factors, such as the performance of an outdoor activity, both during work but also during leisure time on holidays or for the practice of sports or outdoor hobbies. It has to be noted that 20–30 min of outdoor activity in the sun during the hottest hours of the day during summer at intermediate latitudes is sufficient to induce an erythema in pale skin individuals, whereas in winter, many hours could be necessary. Individual behaviors are among the most relevant factors influencing solar UV exposure, such as wearing protective clothes, sunglasses and hats, using sunscreen protections, and shade seeking [4,15,16,17]. These cultural and behavioral factors are fundamental for the prevention of excessive solar UV exposure, and more indications relevant for the prevention of solar UV risk in outdoor workers will be provided in the Section 7. Furthermore, individual factors indirectly influence exposure, but determining a high predisposition for possible UV damage, in particular for the skin, is essential so that people with predisposing conditions are less exposed than others. The most relevant predisposing factor influencing the likelihood of UV damage in humans is photo-type. One of the most applied photo-type classifications is that introduced by Fitzpatrick [18], who identified six different skin photo-types, based on skin pigmentation, ability to tan, and the rapidity of receiving a sunburn. Fitzpatrick photo-types 1 and 2, representing very fair skin, are the most sensitive to UV damage for both acute and long-term effects, but types 3 and 4 are also at risk. Moreover, there are various UV-unrelated skin diseases, such as lupus erithematosus, sclerodermia, and psoriasis, which can be enhanced by UV exposure, so individuals with these pathological conditions need to be more protected when outdoors [3,4,15,16,17].

### 3.3. Occupational Factors

At work, both environmental and individual factors influence acute and long-term (cumulative) solar UV exposure of outdoor workers (OW). For example, the work environment may involve the presence of reflective surfaces, such as water for maritime workers or glass and metal for construction workers. Furthermore, work organization may require workers to perform their activities during the central hours of the day and/or during the hottest seasons, as usually happens both in the construction and agricultural sectors. Also, working posture is relevant in determining the body areas with the highest exposures, such as in agriculture and construction, where different working tasks, such as fruit harvesting from the trees or the ground or the activity of a mason compared to the activity of a tiler may respectively induce a high exposure of the face and chest or the back and nape. Finally, for leisure time, as with for work, the use of individual protection, including adequate clothes, caps, sunglasses, and sunscreen, has a relevant role in determining worker eye and skin exposure [4,15,16,17].

Outdoor work is particularly relevant in influencing cumulative exposure, with possible photochemical damage accumulating in the skin and eyes of the workers for many years, finally resulting in adverse effects. According to the European Agency for Safety and Health at Work, outdoor work is defined as exposed to SR for at least the 75% of their working time, which includes a non-exhaustive list of activities: farmers, silviculturists and horticulturists, farm workers, commercial garden and park workers, postmen and sorters, newspaper delivery workers, physical education instructors, trainers, coaches, and childcare workers [19]. It is estimated that about 15 million workers in Europe are exposed to solar UV; the vast majority (90%) are generally male. UVR is a carcinogen in 36 employment sectors of the European Union, for which 11 rank first among other carcinogens. Also, other occupational diseases statistics, including data from the CARcinogen Exposure (CAREX) database, show that SR is among the first occupational carcinogens, involving at least 10 million exposed workers in Europe [4,19,20].

## 4. Measures and Methods to Evaluate Solar Radiation Exposure in Workers

Solar ultraviolet radiation exposure can be evaluated using different methods. In radiometry, it can be measured by radiant energy and irradiance. Radiant energy is the energy of the electromagnetic radiation emitted by a source into the surrounding environment, measured in Joules. Irradiance is the radiant flux received by a surface per unit area, generally expressed in watts per square meter. However, the effectiveness of solar UV in inducing biological effects varies as a function of wavelength and spectral composition. Therefore, in order to compare exposures with different spectral compositions, and consequently to compare possible risk levels, the effective quantities derived from the previously mentioned radiometric physical quantities are used, called effective irradiance (watts eff/m^2^) and effective radiant exposure (Joules eff/m^2^), respectively [4,16].

A simplified measure of solar UV irradiance on a horizontal plan is represented by the UV index (UVI), which is an estimation of the risk of sunburn for different geographic regions adopted by the World Health Organization (WHO) in 1994. UVI is a linear numeric scale, ranging from 1 to 11+; the higher the value, the greater the potential for skin and eye damage [21,22]. The calculation of UVI is weighted for UV wavelengths to which the skin is most sensitive, according to the CIE action spectrum, so UVI represents a number linearly related to the intensity of sunburns produced by UVR at a specific place. It has also to be considered that the most dangerous UVR bands reaching the skin are in the 295 to 325 nm wavelength range because the vast majority of UVR with shorter wavelengths, even if they have higher skin penetration and damage power, are absorbed by the earth’s atmosphere, in particular by the ozone layer [21,22]. Considering the linearity of the UVI scale, only from a theoretical point of view (under identical meteorological and environmental conditions, with the same person performing exactly the same activity with standard exposing behaviors and identical individual protections adopted), can it be said that the amount of UVR reaching a specific skin site during one hour of exposure to UVI 3 or half-hour exposure to UVI 6 is almost identical.

Considering individual exposure, other quantities with a clinical significance used to evaluate UV exposure are the Minimal Erythemal Dose (MED) and the Standard Erythemal Dose (SED). The MED is defined as the erythemal radiant exposure that produces a just noticeable erythema on a single individual’s previously unexposed skin [4,23]. It is a subjective measure depending on many variables including individual photo-type. The SED is equivalent to an erythemal radiant exposure of 100 J/m^2^ and it is a standardized measure also considering individual sensitivity to the damage proposed by the International Standards Organization (ISO) and the CIE [2,4,24].

These measures of solar UVR are generally applied to evaluate an acute exposure of few hours or a few days. Radiometric physical quantities are obtained with spectroradiometers and dosimeters, evaluating the exposure of an environment where a worker is performing their activity. Often, these meters are worn by the subjects for evaluation of individual exposure, such as polysulphone dosimeters and the more recent electronic dosimeter [4,16].

The abovementioned methods are not adequate for an evaluation of long-term adverse effects related to solar UVR exposure in workers. For these purposes, the large majority of studies currently available in the scientific literature adopted subjective questionnaires, often self-administrated. These surveys usually do not include specific details on the factors influencing exposure, such as on the adoption of preventive measures, and they do not separately consider leisure and occupational solar UVR exposure [4,16].

On the contrary, few studies applied detailed methods to evaluate long-term exposure to solar UVR in groups of OW. Worth mentioning is the study by Rosenthal et al. [25], which presents a model of ocular and facial skin exposure to UV-B that combines interviews on work and leisure activities, and eyeglass wearing and hat usage, with field and laboratory measurements of UV radiant exposure in a group of American water workers. In Australia, McCarty et al. developed a simplified model for quantifying lifetime ocular UV-B exposure considering the ambient UV-B levels, the duration of outdoor exposure, the proportion of ambient UV-B that reaches the eye, and the usage of ocular protection [26].

More recently, Wittlich et al. elaborated a specific algorithm, after a six-month dosimetric measurement campaign in a large group of outdoor workers, obtaining a standardized tool useful for German social accident insurance institutions to calculate and decide whether a sufficient portion of UVR exposure has been accumulated during one’s occupational lifetime [27].

These kinds of methods are important because many individual and environmental factors can modify solar UVR exposure and therefore influence the UV dose able to induce adverse effects at the target organs in outdoor workers.

## 5. Solar Ultraviolet Radiation Exposure Levels in Outdoor Workers

Outdoor workers have very high ultraviolet radiation exposure, usually exceeding the current occupational limits set for artificial UVR exposure, e.g., from the American Conference of Governmental Industrial Hygienists (ACGIH) [28] and from the International Commission on Non-Ionizing Radiation Protection (ICNIRP) [4]. These limits have also been adopted as normative requirements in many areas, such as in Europe according to the European Directive 2006/25/CE on Artificial Optical Radiation. The occupational exposure limit is currently equal to 30 J/m^2^ for a working day (eight hours) for a worker exposed to artificial UV (all UV bands considered, appropriately weighted). This value was initially calculated to prevent photo-keratitis, but can be applied for the protection of both skin and eyes from acute effects [4,28]. When adapting this limit to the CIE erythemal action spectrum, 1–1.3 SED (approximately one-half of a MED for fair skin) would be the limit for eight working hours to protect outdoor workers’ skin from sunburns [2,4,24]. Table 1 presents a review of occupational solar UVR exposure levels in construction, agricultural, and other sectors. We collected data from studies reporting direct measurements at workplace and individual dosimetric measurements, mainly expressed in terms of SED (Table 1). As an example, regarding construction workers, recent studies showed an acute solar UVR exposure of 9.9 SED in Australia [29], a daily dose ranging from 11.9 to 28.6 SED depending on the altitude in Switzerland [30], and an exposure of 6.11 SED in Spain [31]. For farmers, high exposure to UVR have been reported e.g., in New Zealand [32], France [33], Austria [34], and in Tuscany (Italy), where an exposure of the workers’ nape of 14.5 SED was collected in April [35]. With regard to other outdoor workers, in a Spanish study, personal exposure doses of approximately 4 and 11 SED were measured in groups of gardeners and lifeguards, respectively [36]. Lifeguards were investigated in a North American study, experiencing an exposure ranging from 1.7 to 6.9 SED [37].

The measures reported in these studies are useful for evaluating occupational risk due to solar UVR exposure in different professional activities, but they are a measure of acute exposure. Some long-term exposure data could be available for comparison with the leisure exposure of groups of indoor workers (IW). As an example, in Northern Europe, IW receive an annual solar exposure of around 200 SED mainly from weekend and holiday exposure, and principally to the hands, forearms, and face. This value is approximately 5% of the total ambient available. Ocular exposure is rarely relevant, except in case of unusual conditions, such as reflection from snow. At the same latitudes, OW receive about two to three times these exposure doses, so that the annual occupational exposure was estimated to be 400–600 SED per year [4]. These data were recently confirmed in a German study by Wittlich et al., where an annual exposure of 538 SED in an outdoor worker was estimated, and their lifetime occupational exposure was evaluated as 8417 SED using a specific algorithm [27].

## 6. Adverse Health Effects of Solar Radiation Exposure in Outdoor Workers

### 6.1. General Overview of the Adverse Health Effects Related to Solar Radiatio Exposure

Solar radiation exposure may induce several acute and long-term adverse health effects, mainly to the skin and eyes, but the immune system may also be involved, e.g., as found in the reactivation of latent herpes labialis infections. Furthermore, SR exposure may induce positive effects. For example, UV rays have a key role in vitamin D activation and, consequently, in the prevention of diseases such as rickets, osteomalacia, and osteoporosis. Accordingly, insufficient solar UVR exposure is related to a reduction in vitamin D activation. Another beneficial effect of SR exposure is supposed for various psychiatric disorders and possibly for some neoplastic diseases (Table 2), according to the World Health Organization (WHO). WHO published an extensive review of the scientific literature on the health effects of SR in humans in 2006, which are adapted and summarized here in the Table 2 and Table 3. Both adverse and beneficial effects were considered, and the results were classified according to the level of evidence proposed by the WHO and based on the available scientific literature data (Table 2 and Table 3, adapted from WHO [38]). According to the WHO, acute eye effects of SR exposure with strong evidence of causality include photokeratitis and photoconjunctivitis, which can occur in cases with high reflection from surfaces like snow, whereas they are common occupational eye injuries in unprotected workers exposed to artificial UV [39] and solar retinopathy, which is an acute burn of the retina mainly due to the visible and infrared-A components of SR. Chronic diseases of the pterygium include cortical cataract and squamous cell carcinoma (SCC) of the cornea and conjunctiva. Regarding the skin, acute effects with strong evidence of causality are sunburns and photodermatoses; chronic effects include photoaging, actinic keratoses, and other skin cancers such as Basal Cell Carcinoma (BCC), SCC, and Malignant Melanoma (MM) [38].

### 6.2. Long-Term Solar Radiation Exposure and the Eye: Focus on Outdoor Work and Pterygium, Cataract, and Macular Degeneration

#### 6.2.1. Pterygium

The pterygium is a wing-shaped abnormal growth of the conjunctiva onto the cornea that can cause ocular irritation, relevant cosmetic effects, and, in the late stage of the corneal tissue’s invasion, visual impairment. Full knowledge of its pathogenesis is still incomplete, but data indicate a relevant role played by long-term eye exposure to solar UV [38,40,41,42,43,44]. The prevalence of this disease, more frequent in men, is highly variable ranging from 1.1 to 40% in different groups investigated in various places around the world, where the lower the latitude, the higher the prevalence [38,40,41,42,43,44]. According to the WHO, the Population Attributable Fraction (PAF) of pterygium due to solar UVR exposure is 42–74% [38]. We report here a summary from a recent systematic review of the scientific papers published during the period 2008–2017, which specifically considers the role of outdoor work as a risk factor for pterygium [44]. The results of 25 of the 29 studies included in this systematic review are synthetized in Table 4.

This review further supports the role of solar UV radiation in pterygium induction, such as shown by the four-fold higher prevalence in high, very high, and extreme risk areas for the mean annual UV Index (UVI), with a mean pterygium prevalence of 19.3%, versus moderate risk areas with a mean prevalence resulted 4.8%. Considering outdoor work, pterygium resulted in a frequently occurring disease in OW occupationally exposed to SR for many hours during the day and for many years during their working life. Significant odds ratios (ORs) in the reviewed studies were found, ranging from 3.8 in Iran and Ethiopia [52,61] to 1.4 in Taiwan [56]. Considering the UV index, for high, very high, and extremely high-risk areas, we calculated a mean OR of 2.2 based on 12 studies, and the same average OR resulted from five studies of moderate risk areas [44]. Accordingly, OW had increased ORs, ranging from 1.4 to 3.8 for pterygium, and these ORs did not vary significantly with latitude. Occupational exposure to solar UV is one of the most relevant factors associated with pterygium presence, both in the case of high to extremely high levels of environmental UV and in the case of a moderate UV index. Occupational exposure was found to be positively associated with the severity of the disease [53,55,58,70]. Furthermore, in one study, pterygium occurrence was specifically associated with the total number of years worked [51] and in terms of protection, sunglasses, spectacles, and hats were found to be significant protective factors for pterygium in OW [52,54,63].

#### 6.2.2. Cataract

Cataract is currently the primary cause of blindness in the world, responsible for approximately 20 million ongoing cases, and the second most prevalent cause of visual impairment, accounting for approximately 81 million out of 246 million cases [71]. Different risk factors are known for this disease, but one of the main and most diffuse risk factors is long-term UV radiation exposure, for both the UV-A and the UV-B components, absorbed by the lens and acting with a photochemical mechanism [72,73].

Different cataract classifications based on morphological and/or etiological criteria are available, but in epidemiological studies, the most commonly used is the simplified system of three types based on the localization of lens opacities. Nuclear cataract is the most frequent form, followed by cortical cataract, and posterior subcapsular cataract [74,75]. According to the WHO, the upper Population Attributable Fraction of cortical cataract due to solar UVR is 25% [38]. Furthermore, an increasing body of scientific data supports the role of solar radiation in inducing nuclear and posterior subcapsular cataracts [76,77]. Taken as a whole, these data indicate that a reduction in excessive long-term SR exposure could prevent a significant number of visual impairments and blindness worldwide, and to a consequent parallel reduction in medical costs.

Table 5 provides a summary of the results from a recently published systematic review on cataract and occupational SR exposure [77]. Data showed high prevalence of cataracts in OW and a strong association between occupational SR exposure and cataracts. The majority of the studies included in the systematic review showed positive associations between cataracts and outdoor work. Specifically, one longitudinal study found a significant adjusted relative risk in laborers [78], and five studies [79,80,81,82,83] found positive adjusted ORs for cataracts for at least one of the main subtypes, i.e., cortical, nuclear, or posterior subcapsular cataract (Table 5), compared to controls.

Regarding the cataract subtypes, the nuclear form was confirmed to be the most frequent subtype of age-related cataract, followed by cortical cataracts, with posterior subcapsular cataracts being the least frequent. Furthermore, in this systematic review, the major recent evidence for a causal relationship between occupational SR exposure and cataracts were found for the nuclear subtype (Table 5) [77].

#### 6.2.3. Macular Degeneration

Macular degeneration (MD) is a chronic disease affecting the macula, inducing a progressive vision loss, usually starting from the central part of the visual field. The evolution is slow, taking many years to induce an appreciable visual impairment, with different grades of severity from early to late disease stages [92,93,94]. MD is the leading cause of blindness for people over 50 years old in developed countries [95]. According to a multicenter study performed in 2006, the prevalence in Europe is 3.3% [96], and the prevalence is similar in the U.S., where it is estimated that about 10,000,000 people are affected by MD [97].

Chronic retinal damage in MD is considered the result of an alteration in the metabolic sustainment of the photoreceptors cells (rods and cones) and of the retinal pigment epithelium (RPE) related to inflammatory processes and vascular modifications [98,99,100]. Several studies show the accumulation of degradation products in degenerated macular tissue, including lipofuscin granules, which are a result of lipid peroxidation processes and of the oxidation action of oxygen free radicals (OFR), involved in the formation of the “drusen” or body colloids [98,99,100]. OFR formation can be induced by long-term exposure to optical radiation, in particular in the range between 400 and 550 nm wavelength (i.e., near UV-A and blue light). In both laboratory and animal models, near UV and blue light, which are components of the SR spectrum at the Earth’s surface, have proven to induce photochemical damage of the retina [101,102]. Accordingly, long-term SR exposure, as reported by the WHO [38], is a risk factor for MD [97,101,103], together with other factors, such as age, smoking, diabetes, inheritance, and alcohol abuse [92,93,94,104,105,106,107,108]. Therefore, outdoor workers can be considered at risk for the development of this disease.

We report here a summary from a systematic review on the possible occupational risk factors related to an increased risk of MD development [109]. The main results related to occupational SR exposure and MD are synthetized in Table 6. Among other possible occupational risks examined in our review (e.g., chemical exposure), SR exposure of outdoor workers was the most represented, as it was studied in 10 of 13 studies included in the review. All these 10 studies found a positive association between SR exposure and MD. In six studies occupational exposure was evaluated simply by classifying workers as outdoor or indoor [110,111,112,113,114,115], whereas in four studies [116,117,118,119], the exposure was evaluated with a more detailed method, considering subjective and objective data and estimating cumulative SR exposure. We briefly discuss here these latter four studies. In a recent multi-centric European study [118], MD was not found to be associated with current SR exposure, but with both early and late MD, proving an association with a history of past major sunlight exposure more than eight hours outdoor per day, typical of outdoor work, with an OR for early MD of 5.5 (95% CI 1.25–24.6), and of 2.8 (95% CI 1.25–6.2, *p* = 0.01) for late MD. Furthermore, OW were more likely to have late MD with an OR of 2.6 (1.9–3.5), after adjustment for age, sex, and smoking behavior. In another multi-centric European study [117], a significant association was found for subjects, including OW, with the lowest dietary intake of antioxidants and high cumulative blue light exposure in midday hours with an OR of 1.95 (1.1–3.6) for grade 3 MD versus grade 0. History of cumulative exposure to the blue light component of SR was also found to be associated with severe MD (grade 4) in a group of maritime workers, OR = 1.35 (1.0–1.8), whereas no significant association was found for the UV component [119]. These maritime workers were examined in a further study to evaluate a five-year MD incidence. In this case, an increased MD incidence with age and cumulative SR exposure was also shown [116].

Summarizing, current data, despite a low number of published studies on the topic and the rather heterogeneous quality of the studies, support the hypothesis of an association between long-term occupational SR exposure, in particular for its blue-light component, and MD in OW.

### 6.3. Long-Term Solar Radiation Exposure and Skin: Focus on OW and Skin Cancers (Non-Melanoma Skin Cancer, Including Actinic Keratosis and Cutaneous Malignant Melanoma)

The WHO recognizes among the main adverse skin effects related to long term solar radiation exposure as photo-aging, malignant melanoma, and non-melanoma skin cancers (NMSCs), including actinic keratosis (AK) (Table 3) [38]. Notably, AK, according to a recent re-evaluation, can be considered a squamous cell carcinoma (SCC) in situ [120]. Non-melanoma skin cancers (NMSCs), including basal cell carcinoma (BCC), SCC, and AK, are collectively referred to as “keratinocyte carcinomas” [121].

#### 6.3.1. Non-Melanoma Skin Cancers (Including Actinic Keratosis)

NMSCs are the most commonly diagnosed cancers in Caucasians worldwide [122], even if the exact numbers for NMSCs are usually under-estimated due to the lack of registration of these neoplasms in cancer registries. Recent U.S. data estimate that, in 2012, 3.3 million people were diagnosed with an NMSC, but many of these people were diagnosed with more than one NMSC, bringing the total number of NMSC cases approximately to 5.8 million in the U.S. [123]. NMSCs, and in particular BCC, are the most common among all the malignant tumors diagnosed every year in the European population: there are approximately 2,000,000 diagnoses of NMSCs (AK excluded) per year in Europe [5]. In Italy, the incidence of NMSCs is estimated in 119.4 cases per 100,000 male subjects and 90.7 cases per 100,000 women per year, representing a lifetime incidence of 12.5% and 7%, respectively [124]. In the U.S., the yearly cost of treating NMSCs was estimated to be USD $4.8 billion in 2012 [125], whereas in Australia, it was AUD $93.5 million in 2010 [126].

Epidemiological data show high prevalence of AK among Caucasians, both in the northern hemisphere, where it is estimated that 11–25% of the adults develop one or more AKs during their life [127], and in the southern hemisphere. In Australia, approximately 40–60% of adults have one or more lesions [128]. The most relevant risk factors are age, male sex, immunosuppression, a fair photo-type, and the cumulative dose of UVR received from the skin [129,130]. AK frequently progresses in an invasive SCC [131,132]; several studies show that the presence of SCC in association with AK includes a high number of patients, usually higher than the 60% of the samples investigated [133,134]. Furthermore, the recent developments in dermatologic research suggest that AKs are in situ SCCs, rather than pre-cancerous lesions [120].

SCC is less common than BCC, with a ratio between the two types of approximately 1:4, but SCC has more malignant characteristics, with a higher risk of metastasis and related mortality, even if, due to early diagnosis and surgery, the mortality rate has been reduced over the years [135]. In Italy, the estimated age-adjusted SCC prevalence was 0.08% in 2006, indicating approximately 50,000 cases in the Italian population [136]. The SCC incidence increases with age, and accordingly, the vast majority of cases are diagnosed in people over 40 years old [137,138,139]. The diagnosis of SCC produces an additional risk of developing another NMSC in the following three to five years ranging from 18% to 30% [140]. UVR exposure is among the main risk factors for developing SCC [5]: the evidence supporting this is that Caucasian individuals who moved to sub-tropical regions have a higher SCC incidence compared to those living in their country of origin [38]. A concomitant state of chronic immunosuppression contributes to increase the risk of developing SCC in sun-exposed areas [141]. Other risk factors, possibly relevant in relation to occupation, are chemical exposure, such as to arsenic, aromatic hydrocarbons [142,143], and ionizing radiation exposure [142].

The other and more frequent form of NMSC is BCC. In the U.S., it is estimated that more than 2 million people per year are diagnosed with BCC [123]. BCC usually appears in subjects over 60 years old and the prevalence is higher in men, even if recent studies are showing an increasing prevalence in women [144,145]. In Italy, the estimated age-adjusted BCC prevalence was 0.42% in 2006, indicating approximately 250,000 cases in the Italian population [136]. As for SCC, long-term solar UVR exposure is a recognized risk factor for the development of BCC as well as fair photo-type [5,38], but in this case, intermittent intense UVR exposure is particularly relevant [145]. Genetic risk factors also have to be considered for the development of BCC, in particular in the case of multiple presentation, such as immunosuppression, photosensitizing drug use, human papillomaviruses infections, smoke, chemicals, and ionizing radiation exposure [145].

In evaluating the relationship between the two main types of NMSCs and occupational solar UVR exposure, according to the WHO, “the risk of BCC increases with increasing occupational exposure, but particularly with increasing non-occupational or intermittent exposure to the sun”, whereas for SCC there is a “convincing” epidemiologic and biologic evidence of a causal association with UVR exposure. The risk is related to total lifetime sun exposure, “but particularly occupational sun exposure” [38]. Two specific systematic reviews and meta-analyses on the association between occupational solar UVR exposure and SCC and BCC were recently published [146,147]. Considering SCC, Schmitt et al. [146] showed that 89% (16 studies) of the articles included in their systematic review found an increased risk of SCC in individuals with an occupational solar UV exposure compared with individuals without occupational solar UV exposure. A statistical significance was reached in 12 of the studies included, whereas only in two studies was no association reported. At the meta-analysis phase, a pooled OR of 1.8 (95% CI 1.4–2.2) was calculated, similar in cohort and in case-control studies. Notably, according to the authors, the pooled OR was, most likely, substantially underestimated due to the qualitative UV exposure assessment performed in the majority of the studies reviewed. Finally, the authors reported an increasing strength in the association between occupational solar UV exposure and SCC risk with decreasing latitude [146]. Considering BCC, the meta-analysis of Bauer et al. [147] revealed a 40% increase in the risk of developing cancer in outdoor compared to indoor workers. In this case, the authors suggested that the observed pooled OR of 1.4 (95% CI 1.2–1.7) calculated at the meta-analysis phase should substantially under-estimate the true relationship between occupational solar UV exposure and BCC risk [147].

#### 6.3.2. Malignant Melanoma

Malignant melanoma incidence has increased constantly worldwide in recent years, and by an average of 3–4% every year in the U.S. [148] over the last 40 years. Moreover, the incidence of melanoma in fair skinned individuals (e.g., Fitzpatrick phototypes 1–3) increases at low latitudes: according to the results of the Global Burden of Diseases (GBD) project, the global incidence of melanoma in 2015 was 351,880 cases, with the highest rates in Australia and New Zealand, with both at 54 per 100,000 per year, followed by the European countries of Norway and Sweden (26 per 100,000/year) and the Netherlands (25 per 100,000/year). The lowest incidence was observed in Southeast Asia and in particular in the Pacific area, with rates ranging between 0.7 and 1.4 per 100,000 people per year [149]. Considering the melanoma mortality, according to GBD estimates, the number of deaths per year attributable to melanoma in the world are approximately 50,000, higher in men [149].

UV exposure is among the most relevant risk factors for melanoma, together with the number of melanocytic nevi, familial history, and genetic susceptibility [150]. The increase in environmental UV radiation is strongly associated with a high risk of melanoma: as ozone levels are depleted, the atmosphere increasingly loses its protective filter function and more solar UVR reaches the earth’s surface [151]. The WHO estimates that a 10% decrease in ozone will result in an additional 4500 melanoma cases per year [38].

Considering the association between melanoma and long-term UV exposure, which is typical of outdoor work, various signs of chronic exposure, as in BCC and AKs, have been significantly associated with melanoma occurrence [5,152,153]. Nevertheless, the pattern of UV exposure typically associated with a higher melanoma risk is that of intermittent sun exposure, including repeated sunburns, especially in younger ages and childhood [5,152,153]. On the other hand, despite the association with chronic actinic damage, poor associations have been found with regard to cumulative sun exposure and long-term sun exposure in general [5,152,153].

Examining the risk for outdoor workers, in its monograph, the IARC concluded a “…lower-than average phenotypic risk for skin carcinogenesis among outdoor workers”, recognizing the association with intermittent exposure typical of leisure time. In the WHO “Environmental Burden of Disease Series, No. 13”, only 8 of the 49 reviewed studies on the association between solar UVR exposure and melanoma considered occupational sun exposure, and only one [154] found a positive significant relative risk of 6.01 (CI 95% 2.08–17.36) for outdoor work versus indoor work [38].

In conclusion, scientific literature data indicate an overall lack of evidence for a raised incidence of melanoma in outdoor workers [5,38,153], whereas some studies suggest an increased melanoma risk for indoor workers. A possible explanation for this apparently paradoxical observation is that specific groups of indoor workers (e.g., managers, physicians, etc.) have a high economic income compared to OW, so that they may spend longer vacation periods in places with higher UV indexes. Moreover, because of the lower UV exposure of indoor workers during the year, they are usually not tanned when starting their holidays, whereas tanning acts as sunburn protection for OW. These possible scenarios suggest that indoor workers are more likely to report sunburns and intense intermittent UV exposure during leisure time compared to OW. These events may induce a possible increased risk of melanoma [153]. Another possible explanation to be considered is the so-called “healthy worker effect”: workers with a very fair photo-type or with other relevant risk factors for melanoma (e.g., with melanoma cases within their families) may be less likely to be employed in outdoor occupations.

## 7. Prevention of Solar Radiation Exposure Risk in Outdoor Workers

The prevention of skin and eye adverse health effects in outdoor workers exposed to solar UV needs to be based on various preventive actions, including collective technical and organizational interventions, information and specific training of workers, use of individual protective equipment, change in individual behaviors, and adequate health surveillance of exposed workers [3,4,15,16,17,21,22]. Nevertheless, these preventive measures are currently inadequately applied and the major reason for this would be the lack of standards, legislation, and recommendations for the prevention of this occupational risk. To our knowledge, among the most relevant and internationally recognized experiences for the prevention of UV risk at work, there are the documents from ICNIRP [4] and ACGIH [28], proposing specific occupational exposure limits, applicable for artificial optical radiation. However, given the highly variable environmental solar UVR levels as well as behavioral effects and different exposure geometry, the application of these limits to solar UV exposure is not practical [155]. Accordingly, new efforts to enforce the protection of OW should be desirable, at both a legislative and political level. To raise awareness of the issue of occupational UV risk for OW, the reporting of occupational SR-related diseases, and in particular skin cancers, should be extremely relevant [17,156]. Among the main examples of preventive interventions that can be applied to reduce the adverse health impact of solar UV risk there are four main techniques:

(1) Collective Technical/Organizational Measures. These include artificial or natural coverage and shading of the workplace; organization of specific indoor breaks, or at least breaks in UV-shielded areas during work and lunch, to reduce exposure during the central hours of the day; re-organization of working activities in order to avoid (or limit) outdoor work during the central hours of the day (e.g., organizing specific work activities in UV shielded areas, starting work earlier in the morning, prolonging lunch breaks, etc.), especially in the periods of the year with the highest UV indexes [3,4,15,16].
(2) Health and safety information and training. As the risk of developing adverse effects increases with age and with the cumulative UV dose received, training and information activities should be implemented as early as possible, possibly in all schools preparing for outdoor professions and all outdoor workers. Contents of these prevention initiatives should include the mechanisms and effects of acute and chronic SR exposure, he possible preventive measures to be adopted, as well as the importance of auto-examination and health surveillance, and of periodic dermatologic and ophthalmologic examinations. The adoption of preventive materials as fact-sheets and signals, recommending, for example, to possibly avoid exposure when UVI is higher than 3 and reminding workers to protect themselves adequately with clothing, hats, UV filtered eyeglasses, and sunscreen, can be suggested. Information on the reflection indexes of the surrounding surfaces should be important, especially for the protection of the eye [3,4,15,16,17,21,22].
(3) Personal protection. Sunglasses for occupational use must fulfill standard requirements in terms of both filtering power of the lenses and shape of the sunglasses. They have to be marked, reporting their technical characteristics, and their lenses need to be adequately large and adherent, with large lateral bars. Appropriate clothing should include long-sleeved shirts and trousers from light-proof fabrics (cotton wool or synthetic fibers) with high Ultraviolet Protective Factors (UPF: recommended 50+). Adequate headgear would include broad-brimmed helmets or broad-brimmed hats supplied with sun shields and neck guard. Waterproof sunscreens (recommended SPF 50+) must be applied on all uncovered skin areas (and under the clothes if they do not have a sufficient UPF). An abundant application at least 20 min before exposure is recommended, as well as frequent re-applications. A new application every two hours is recommended when UVI is above 3 [3,4,15,16,17,21,22].
(4) Health surveillance. Occupational physicians should perform pre-employment medical examinations and periodic medical examinations of outdoor workers to adequately prevent adverse long-term effects to eyes and the skin. Of particular relevance is the recognition of individual conditions inducing a particular sensitivity to UV risk, such as photo-type 1 or 2, prolonged use of photosensitive drugs, wounds, suspected skin lesions, and presence of UV sensitive skin diseases (e.g., psoriasis). For the eye, lens opacities, corneal lesions, etc., would induce photosensitivity. A collaboration with dermatologists and ophthalmologists, or with other specialists, would be useful in specific cases. When UV-related diseases are diagnosed in outdoor workers, reporting these diseases to the compensation authorities is crucial, not only for the legal recognition of the disease for the individual worker, but also to uncover the real dimension of the emerging issue of UV-related occupational diseases and for the development of better prevention of this underestimated work-related risk [3,4,15,16,17].

## 8. Conclusions

In this review, we presented a comprehensive overview of the main relevant aspects of the risks related to occupational solar radiation exposure, focused in particular on the UV component. As a conclusion, we summarize here some main points to be considered for a better protection of outdoor workers.

Photo-chemical UVR damage to the eyes and skin is cumulative, and consequently, repeated exposure over the years, even if quite low, may result in disease. Accordingly, it is always important to implement preventive measures aiming to reduce UV exposure, from an early age, even in the case of moderate UV index. Excessive exposure is responsible for a significantly increasing risk of relevant adverse health effects, in particular to the eyes and skin, including pterygium, cataract, macular degeneration, and melanoma for the eye; and keratinocytes carcinomas and malignant melanoma for the skin. Notably, for cutaneous malignant melanoma, despite being recognizes in UV exposure as a major risk factor, to date no increase in the risk for outdoor workers has been shown in scientific studies.

Application of UV occupational exposure limits to solar UV involves many practical issues, but the scientific literature shows that outdoor workers frequently considerably exceed current ACGIH and ICNIRP limits. So occupational solar UVR exposure should be reduced to the lowest level possible, as it is carcinogenic to humans (e.g., group 1 IARC). Despite the frequency of SR-related adverse health effects in outdoor workers, these diseases are under-reported to the national legal and workers’ compensation authorities. This is particularly true for skin cancers, which are the most frequent cancers in Caucasians, but are scarcely reported as occupational diseases. An increased reporting of solar UVR-related occupational diseases should be a fundamental step in raising public awareness and promoting the adoption of preventive measures.

A reduction in the occupational risk related to solar radiation exposure is possible. It can be based on: (1) collective measures including specific legislation, standards, and recommendations, workers’ education and training, and preventive technical and organizational interventions; (2) individual measures including adoption of personal protective equipment and promotion of appropriate individual behaviors; and (3) secondary prevention, which includes health surveillance of outdoor workers performed by trained physicians.

## Figures and Tables

**Table 1:** Direct measurements of solar ultraviolet (UV) exposure in groups of outdoor workers in different sectors. Results of the measurements are in Standard Erythemal Doses (SED) per day.

	Reference	Population, Month/Season, Place	Results of the Measurements (SED/day)
**Construction Sector**	[33]	126 workers, Summer, France	10.1
	[29]	493 OW September–November, Queensland (North Australia)	Pavers–Tilers 10
			Dogger 8.3
			Roofers 7.6
			Fencers 6.2
			Plant operators 3.1
			Painters 1.1
			Cabinet makers 0.3
			Laborers 5.9
			Steel fixers 5.6
			Inspectors 2.5
			Concreters 4.7
			Bricklayers 4.7
			Supervisors 3.4
			Carpenters 5.3
			Riggers 6
			Plumbers 5.7
			Other Workers 4.9
			All workers 4.5
	[32]	77 OW: 39 construction and 19 road workers, Summer (December), New Zealand	5.25 for construction workers 5.31 for road workers
	[30]	20 workers, Switzerland, July–September, at three different altitudes: plain (500–600 m); middle (1400–1500 m); high mountain (2000–2500 m)	11.9 in plain 21.4 at middle altitude 28.6 in high mountain
	[31]	8 workers, Valencia, Spain	6.11
**Agricultural Sector**	[33]	23 Gardeners and 108 farmers, Summer, France	12 for gardeners 9.5 for farmers
	[32]	77 OW, of which 16 horticulturists, Summer (December), New Zealand	5.61
	[35]	31 vineyard workers, April, July, October, Tuscany, Italy	April: Nape = 14.5; Arm = 10.3 July: Nape = 10.0; Arm = 5.9 October: Nape 3.0; Arm 2.0
	[34]	12 farmers, April and October, Austria	2.99
	[36]	4 gardeners, June–July, Valencia, Spain	4.1
**Other Occupational Sectors**	[33]	741 workers with various occupations (not all OW)	Cultural, art, social workers 9.2 Industrial workers 7.9 Telecommunication workers 7.9 Transporters & mail carriers 7.7 Office workers 7.3 Commercial & service agents 6.9 Managers 6.3 Protective services workers 6.2 Engineers, researchers 6.1 Health professionals & personal care workers 6.0 Leisure and sport workers 5.9 Shopkeepers 5.4 Cleaners and service workers 4.9 Restaurant workers 4.6 Teaching professionals 3.5 Child care workers 3.3
	[37]	168 lifeguards, June–July in: (1) <35° N (Arizona, Texas); (2) >40° N (Nebraska, Oregon, USA)	South US 3.3 (Texas) 3.2 (Arizona) North US 6.2 (Nebraska) 1.7 (Oregon) Mean (all sites) = 3.3
	[36]	5 lifeguards, summer (June–July), Valencia, Spain	11.4

**Table 2:** Beneficial effects on diseases of solar radiation exposure (WHO 2006, adapted) [38].

Target	Diseases
Bone system	Rickets, osteomalacia, and osteoporosis depending on reduced vitamin D production *
Cardiovascular system	Hypertension
Lymphopoietic system	Non-Hodgkin lymphoma
Prostate	Cancer
Breast.	Cancer
Colon	Cancer
Psychiatric disorders	Seasonal affective disorder; Schizophrenia; General well-being.
Others	Rheumatoid arthritis, Type 1 diabetes, Multiple sclerosis (for the immunomodulating role of solar UV); Tubercolosis (for the regulatory role of solar UV in Vitamin D production)

* Effects with strong evidence of causality according to WHO.

**Table 3:** Adverse health effects of solar radiation exposure in humans (WHO 2006, adapted) [38].

Target	Adverse Health Effects Caused by Excessive SR Exposure
Immune system	Acute: Reactivation of latent viral infection—herpes labialis *; suppression of cell-mediated immunity; increased susceptibility to infection; impairment of prophylactic immunization
	Long-term: Reactivation of latent viral infection—papillomavirus
Eye	Acute: photokeratitis and photoconjunctivitis *; solar retinopathy *
	Long-term: Pterigyum *; cataract: cortical *, nuclear, and sub-capsular; climatic droplet keratopathy; Pinguecula; melanoma; macular degeneration; corneal and conjunctival squamous cell carcinoma *
Skin	Acute: Sunburn *; Photodermatoses *
	Long-term: Photo-ageing *; actinic keratosis *; basal cell carcinoma *; squamous cell carcinoma *; cutaneous malignant melanoma *; cancer of the lip

* Effects with strong evidence of causality according to WHO.

**Table 4:** Mean pterygium prevalence and mean odds ratios (ORs) calculated based on scientific studies on pterygium and outdoor work published in the period of 2008 to 2017 according to the UV risk areas: evaluation of the differences (adapted from Modenese and Gobba [44]).

UVI Risk Area	Reference	Pterygium Prevalence (%)	Average Pterygium Prevalence Per Area (%)	OR (95% Confidence Interval) for Pterygium and Occupational Exposure to SR vs. No Occupational Exposure	Mean OR in Risk Area
**UVI ≤ 5 Moderate Risk**	[45]	/	4.8	3.1 (1.9–4.8)	2.2
	[46]	2.5		1.8 (1.2–2.6)	
	[47]	6.2		1.5 (1.1–1.9)	
	[48]	7.1		/	
	[49]	5.9		2.3 (1.0–5.0)	
	[50]	2.5		2.3 (2.5–5.4)	
**UVI ≥ 6 High Risk**	[51]	13.3	19.3	/	2.2
	[52]	38.7		3.8 (2.2–6.5)	
	[53]	10.1		2.1 (1.1–4.0)	
	[54]	9.5		/	
	[55]	12.3		2.2 (1.1–4.5)	
	[56]	25.2		1.4 (1.2–1.9)	
	[57]	13		/	
	[58]	52		/	
	[59]	/		2.5 (1.3–4.9)	
	[60]	11.9		/	
	[61]	/		3.8 (1.0–14.7)	
	[62]	11.7		1.8 (1.5–2.2)	
	[63]	19.3		1.5 (1.1–2.2)	
	[64]	9.4		/	
	[65]	10.9		2.2 (1.2–4.1	
	[66]	30.8		1.8 (1.3–2.5)	
	[67]	4.4		1.5 (0.9–2.3)	
	[68]	16.2		/	
	[69]	39		1.5 (1.1–1.9)	

**Table 5:** Cataract prevalence and mean ORs calculated in scientific studies conducted in outdoor workers (OW) in the period of 1998 to 2017 (adapted from Modenese and Gobba, 2018) [77].

Reference	Cataract Prevalence (%)	OR (95% CI) for Cataract and Occupational Exposure to Solar Radiation vs. No Occupational Exposure/Cataract Subtype Associated (If Investigated)	Notes/Other Results
[84]	33.2	No association	Higher education level vs. lower OR = 0.6 (0.4–0.9)
[85]	40.4	0.9 n.s.	/
[86]	40.1	/	Significant higher prevalence in male OW
[61]	25.4	/	Prevalence in a group of quite young Indian salt workers
[87]	/	1.8 (1.5–2.9) in urban context 5.9 (4.8–6.9) in rural context	Unadjusted OR
[88]	29.8	/cortical	Prevalence in a group of French mountainers
[78]	/	/cortical	Longitudinal study: Relative Risk in laborers = 2.2 (1.03–4.9)
[79]	36.8	2.9 (1.1–7.6)/nuclear	/
[80]	/	3.7 (1.5–9.0)/nuclear	OR 3.2 (1.2–8.2) when considering the use of protective equipment
[81]	36.3	1.1 (1.0–1.2)	/
[89]	/	/	OR 5.33 (1.7–16.7) for OW with catarct and polymorphism of glutathione S-transferase M1 gene vs. OW from the control group and same gene expression
[82]	/	1.8 (1.1–2.8)/nuclear 2.8 (1.4–5.7)/posterior subcapsular	/
[90]	/	2.75 (1.5–4.5).	Unadjusted OR
[83]	25.8–37.2	2.6 (1.45–4.67)	/
[91]	/	/	OR 2.7 (1.2–6.3) for OW with cataract and NQO1 C609T gene polymorphism vs. OW from control group without the polymorphism

n.s. = not significant; OR = odds ratio; OW = outdoor workers.

**Table 6:** Macular degeneration (MD) prevalence and mean ORs calculated in scientific studies conducted in OW (adapted from Modenese and Gobba, 2018 [109]).

Reference, Location	Main Results: Association between Occupational SR Exposure and Macular Degeneration
[116], USA	Five-year incidence in Maritime workers = 50–59 years: 7%; 60–69 years: 14%; >70 years: 26%. Cumulative exposure to SR = 0.84 ± 0.63 Maryland Sun Years
[110], Croatia	OW vs. controls: 70 vs. 30% (X^2^ = 17,633, *p* < 0.0001)
[117], Europe	Subjects with lowest dietary intake of antioxidants and high blue light exposure in central hours of the day = OR 3.7 (95% CI 1.6–8.9) for neo-vascular MD vs. atrophic; OR 1.9 (95% CI 1.1–3.6) for MD grade 3 vs. 0
[111], Croatia	Three-year incidence = 1.9% in OW vs. 0.8% in indoor workers (*p* < 0.001)
[112], Croatia	113 fishermen, sea workers, and farmers with SR exposure > 8 h/day had MD, X^2^ 186.22, *p* < 0.001
[113], Iran	Gene XRCC7 polymorphism in OW: OR 3.1 (95% CI 1.04–9.4; *p* = 0.04)
[118], Europe	Prevalence = 20.3% early MD; 31.9% late MD OR: 2.6 (95% CI 1.9–3.5) after adjustment for age, sex, and smoking behavior for late MD and OW; n.a. with early MD Considering past SR exposure > 8 h/day: OR = 5.5 (95% CI 1.25–24.6) for early MD OR = 2.8 (95% CI 1.25–6.2) for late MD
[119], USA	MD grade 4 in maritime workers = 1.2% OR = 1.35 (95% CI 1.0–1.8) for blue light exposure, n.s. for UV
[114], Nepal	Composition of the sample = 42.6% farmers (most represented occupational group, *p* = 0.077)
[115], Croatia	Two-year incidence = 18% in OW (farmers and fishers) vs. 2.5% in indoor workers

CI = Confidence Interval; MD = Macular Degeneration; OR = odds ratio; OW = outdoor workers; SR = solar radiation.
